# Impact of Food Additives on Gut Homeostasis

**DOI:** 10.3390/nu11102334

**Published:** 2019-10-01

**Authors:** Federica Laudisi, Carmine Stolfi, Giovanni Monteleone

**Affiliations:** Department of Systems Medicine, University of Rome Tor Vergata, 00133 Rome, Italy; federica.laudisi@gmail.com (F.L.); carmine.stolfi@gmail.com (C.S.)

**Keywords:** Western diet, microbiota, dysbiosis, IBD, emulsifiers, maltodextrin, colitis

## Abstract

In physiological conditions, the gut is heavily infiltrated with various subsets of inflammatory cells, whose activity is tightly controlled by counter-regulatory mechanisms. Defects in such mechanisms can favour the development of chronic intestinal disorders, such as Crohn’s disease (CD) and ulcerative colitis (UC), the principal forms of inflammatory bowel diseases (IBD) in humans, as well as systemic disorders. Over the last years, the frequency of intestinal and systemic immune-inflammatory disorders has increased in previously low incidence areas, likely due to the Westernization of lifestyles, including dietary habits. The Western diet is characterized by high consumption of proteins, saturated fats and sweets, as well as by a broad use of food additives (e.g., emulsifiers, bulking agents), which are used to preserve and enhance food quality. Accumulating evidence suggests that food additives can perturb gut homeostasis, thereby contributing to promote tissue-damaging inflammatory responses. For instance, mice given the emulsifiers carboxymethylcellulose and polysorbate 80 develop dysbiosis with overgrowth of mucus-degrading bacteria. Such an effect triggers colitis in animals deficient in either interleukin-10, a cytokine exerting anti-inflammatory and regulatory functions, or Toll-like receptor 5, a receptor recognizing the bacterial flagellin. Similarly, the polysaccharide maltodextrin induces endoplasmic reticulum stress in intestinal goblet cells, thereby impairing mucus release and increasing host susceptibility to colitis. In this review, we report and discuss the current knowledge about the impact of food additives on gut homeostasis and their potential contribution to the development of inflammatory disorders.

## 1. A Complex and Fine Balance: Gut Homeostasis

In healthy individuals, the intestine contains the largest population of immune cells in the body and this reflects the fact that it has a large surface area continuously exposed to dietary antigens and microorganisms [1]. This state of “physiological” inflammation has the challenge of promptly responding to pathogens without inducing morphological/functional gut alterations [2]. Complex regulatory pathways must therefore maintain intestinal immune homeostasis in a healthy intestinal tract [2]. The epithelium represents a highly selective barrier between the underlying lamina propria and the external environment [3]. Each intestinal epithelial cell maintains strict contact with its neighbours and seals the surface of the gut with tight junctions [3]. Additionally, the gut epithelium contains goblet cells, which secrete mucus [3,4], and Paneth cells, which synthesize antimicrobial peptides (i.e., lysozyme and defensins) [5]. Nevertheless, the gut epithelial barrier does not completely prevent luminal antigens from entering the tissues, and few gut bacteria and bacterial products/components can be detected in the mesenteric lymph nodes draining the normal gut [6]. Luminal antigens can also reach the gut immune cells through the follicle-associated epithelium, which overlies the organized lymphoid tissues of the intestinal wall (i.e., Peyer’s patches and follicles) [7]. In addition, mucosal dendritic cells can extend processes between gut epithelial cells into the lumen and sample commensal and pathogenic bacteria [8,9]. Despite luminal antigens interact with the intestinal immune system, excessive adaptive responses are prevented by additional counter-regulatory mechanisms, which include induction of programmed T cell death by apoptosis, lymphocyte suppression by regulatory CD4^+^ T cells, regulatory macrophages/dendritic cells, and immune-suppressive cytokines [2]. Accumulating evidence suggests that perturbations in any of these regulatory mechanisms facilitate the establishment of a pathological process that, under specific genetic and/or environmental conditions, can result in the development of inflammatory bowel diseases (IBD) or systemic inflammatory disorders [10,11].

The main IBD in human beings are Crohn’s disease (CD), in which the inflammatory, segmental and transmural lesions can involve any part of the gastrointestinal tract, and ulcerative colitis (UC), which is characterised by a mucosal inflammation of the rectum and colon [10]. The aetiology of both IBD is unknown even though there is evidence that both disorders are triggered by an exaggerated immune response against intestinal commensal bacteria in genetically susceptible individuals as a result of the action of many environmental factors [12,13], including dietary factors [14,15,16,17]. IBD are more common in industrialized countries [18,19,20], but in recent years a rapid increase in IBD frequency has been reported in previously low-incidence areas (e.g., Asian countries), concomitantly with the acquisition of Westernized dietary habits [21,22,23]. It has been hypothesized that the Western dietary factors associated with the development of metabolic syndrome and obesity could contribute to the onset and progression of IBD [16,24]. For instance, increased intake of saturated fatty acids can drastically change the intestinal microbiota composition, leading to the overgrowth of specific pathogens involved in IBD development [25,26]. Indeed, mice given fat-enriched diet exhibited changes in bile acid composition, resulting in the overgrowth of the pathogenic bacterium *Bilophila Wadsworthia* which, in turn, can trigger intestinal inflammation [25]. Dietary compounds can also exert a detrimental effect on the intestinal epithelial barrier by triggering endoplasmic reticulum stress (ERS), a phenomenon activated upon exposure to low glucose levels, hypoxia and accumulation of misfolded proteins [27]. Such a mechanism promotes the unfolded protein response (UPR), which attenuates mRNA translation and promotes protein folding, thus re-establishing normal endoplasmic reticulum function. However, persistent activation of ERS may alter epithelial cell activity and impair the barrier function. Western diet can also induce oxidative responses in the gut epithelium and decrease expression of tight-junction proteins or mucus layer, with the downstream effect of facilitating bacteria translocation into the lamina propria [27,28]. More recent studies have shown that food additives, which are commonly present in Western processed foods, can exert deleterious effects in the gut. In this article, we review the available data on the impact of food additives on gut homeostasis and their contribution to the development of gut and systemic pathologies.

An extensive systemic literature review was conducted using the following keywords: inflammatory bowel disease, gut dysbiosis and food additives. The literature search included original articles from 1993 to 2019.

## 2. Food Additives: Properties, Applications and Legislation

Food additives are substances commonly used during food processing to increase shelf life and improve quality and taste of pre-packaged foods. Food additives can also be used as stabilizers, coating or filler agents and their presence in food products is often indicated with a unique “E-number” [29]. The European food additive market is expected to increase in the next decades, assuming a key role in the food-related economy. Asian food additive market is now experiencing a rapid growth as well, following in the footsteps of the Western food market’s trend [30]. The use of additives in food processing is regulated by specific laws and must be authorized by the Food and Drug Administration (FDA) in the United States and the European Food Safety Authority (EFSA) in the European community [31]. However, most food additives currently present on the market were approved in the ’70s–’80s following inadequate in vitro and in vivo experimentation [32]. This outdated safety evaluation, together with the increase of the food additive market volume, suggests the need of further assessment of the potential detrimental effects of these substances on human health, especially for those subjects with intestinal or systemic disorders (e.g., IBD patients, metabolic syndrome patients) or increased susceptibility to pathogenic conditions (e.g., relatives of colon cancer patients, relatives of IBD patients).

## 3. Effects of Food Additives on Gut Homeostasis

Many pre-clinical studies have recently linked the increased and prolonged consumption of food additives with the development and progression of various forms of colitis, colorectal cancer, and metabolic syndrome, which is characterized by increased adiposity, dysglycemia, and low-grade intestinal inflammation (Table 1).

### 3.1. Emulsifiers

Chassaing et al. reported the detrimental effects of a long-time exposure to low doses of the dietary emulsifiers polysorbate 80 (P80) and carboxymethylcellulose (CMC), two detergent-like molecules that are incorporated into most processed foods to improve texture and stability, on intestinal homeostasis in mice [33]. The authors observed an alteration of the microbiota characterized by overgrowth of mucus-degrading bacteria. Moreover, they reported the development of colitis in animals deficient in either interleukin-10, a cytokine exerting anti-inflammatory and regulatory functions, or Toll-like receptor 5, a receptor recognizing the bacterial flagellin. On the other hand, wild-type mice given orally the two emulsifiers exhibited symptoms similar to those occurring in patients with metabolic syndrome [33]. Notably, germ-free mice receiving fecal transplantation from emulsifier-treated animals developed changes in microbial composition and low-grade inflammation, further supporting the role of P80 and CMC in promoting dysbiosis-driven pathologies [33]. Mice protractedly fed with P80- and CMC-enriched diet (i.e., 141 days) developed a low-grade intestinal inflammation, ultimately resulting in the development of neoplastic lesions in the colon [34]. An additional study from the same group documented the direct effect of the two emulsifiers on microbiota [35]. By using the mucosal simulator of the human intestinal microbial ecosystem (M-SHIME) model, which maintains a complex stable human microbiota in the absence of a live host, the authors showed that both P80 and CMC acted directly upon human microbiota leading to the expansion of pro-inflammatory pathogenic bacteria [35]. Emulsifier-treated M-SHIME microbiotas triggered low-grade intestinal inflammation once transplanted into germ-free mice thus recapitulating many of the host/microbial alterations seen in mice given directly the emulsifiers. [35]. P80- and CMC-dependent changes in the intestinal microbiota were also responsible for sex-specific behavioral and neural alterations in mice [36]. In particular, females acquired an anti-social behavior, whereas males showed increased levels of anxiety [36]. Interestingly, both alterations correlated with distinct changes in the microbiota profile and in the levels of agouti-related peptide and α-melanocyte stimulating hormone, two neuropeptides involved in the modulation of anxiety-related behavior, appetite and energy [36]. These latter findings suggest that dietary emulsifiers may also have an impact on the gut-brain axis and induce psychological/behavioral disorders in the exposed subjects through a microbiota-dependent mechanism.

### 3.2. Coating and Thickening Agents

Maltodextrin (MDX), a polysaccharide produced through the enzymatic and chemical degradation of corn, potato or rice starch, is commonly used as filler and thickening agent in food processing. Accumulating evidence suggests that MDX can impair gut homeostasis via multiple mechanisms and promote intestinal pathologies [37,38,39,40]. For instance, MDX has been involved in the development of necrotizing enterocolitis (NEC), a severe intestinal disorder of the newborns [37]. Thymann and colleagues orally exposed preterm pigs to either MDX or lactose (sham) by orogastric feeding tube for 36 h or until NEC development [37]. Animals given MDX had higher incidence of NEC, characterized by significant body weight loss, mucosal villus erosions and inflammation, and dysbiosis [37]. MDX was also reported to affect the intestinal microbiota and promote the outgrowth of pathogens [38,39]. Nickerson and McDonald showed that MDX favors the adhesiveness of the CD-associated adherent and invasive *E. coli* (AIEC) strain LF82. AIEC strains have been isolated from CD patients with ileal lesions and supposed to play a pathogenic role in this disorder [38]. As AIEC strains carry the virulence factor MalX, which is a MDX-binding component of the maltose/MDX metabolism system, and MDX-grown LF82 form robust biofilms, it is tempting to speculate that MDX metabolism may help colonization of *E. coli* in the terminal ileum [38]. The same researchers showed that oral MDX impaired anti-bacterial response of mice to *Salmonella* infection and increased bacteria mucosal colonization through alterations of the intestinal epithelial barrier [39]. Another piece of evidence supporting the negative effect of MDX on gut homeostasis comes from our recent study [40]. Mice receiving MDX for 5 weeks in drinking water exhibited MAP-kinase-driven ERS activation in goblet cells, which was accompanied by decreased mucus production and increased susceptibility to colitogenic stimuli [40]. Interestingly, MDX did not apparently perturb the composition of mucosa associated-microbiota, suggesting that, at least in this model, MDX-induced detrimental effects on intestinal epithelial cells were microbiota-independent [40]. We also showed that long-term oral intake of MDX (i.e., 10 weeks) promoted a low-grade intestinal inflammation in mice, with alterations of colonic morphology and increased expression of the inflammatory markers IL-1β and lipocalin-2 [40].

### 3.3. Non-Caloric Artificial Sweeteners (NAS)

Non-caloric artificial sweeteners (NAS) are common food additives that provide sweet taste and a low-caloric content. NAS are largely present in soft drinks, snack foods and dairy products [58]. Suez et al. showed that long-term oral intake of saccharin, a commonly used NAS, induced glucose intolerance and dysbiosis in mice [41]. Treatment with antibiotics protected animals from the disease, thus confirming the important role of NAS-dependent intestinal dysbiosis in driving such metabolic abnormalities [41]. Notably, both short-term (i.e., 7 days) and long-term NAS consumption caused intestinal dysbiosis and altered glycemic response even in human subjects [41]. NAS-dependent microbiota alteration associates also with changes in bacterial fecal metabolites. In this context, Bian and colleagues observed that 6-month consumption of sucralose in drinking water in mice resulted in altered host microbiota and related metabolites, in particular the ones belonging to the tryptophan metabolism (i.e., quinolinic acid and kynurenic acid) [42]. Tyrosine, as well as its metabolites p-hydroxyphenylacetic acid and cinnamic acid, which are known to restrain reactive oxygen species (ROS) production, and bile acids were also impaired following sucralose-enriched diet. Moreover, such alterations contributed to trigger and sustain liver inflammation in the exposed mice [42]. Moreover, Oliver-Van Stichelen and colleagues showed that pre-natal exposure to sucralose and acesulfame-K, another artificial sweetener, altered both microbiota composition and related metabolites of mouse pups, leading to an impairment of their hepatic detoxification mechanisms [43].

Another FDA-approved artificial sweetener is neotame, which is chemically related to aspartame, but more efficient in providing sweet taste. Neotame use can change gut microbiota composition and reduce α-diversity in mice [44]. Additionally, neotame oral intake by gavage can change host metabolic pathways and increased cholesterol and lipid secretion in feces at the expense of malic and glyceric acids [44].

SAMP1/YitFc (SAMP) mice spontaneously develop ileitis showing morphological and immunological similarities with CD. Such mice exhibit exacerbated mucosal inflammation following intake of the artificial sweetener Splenda [45]. Six-week-intake of Splenda in drinking water, at the maximum dose recommended by the FDA (i.e., 3.5 mg/mL) promoted gut microbiome dysbiosis and accumulation of *Proteobacteria* in both SAMP mice and its parental ileitis-free control mouse strain AKR/J (AKR) [45]. However, a significant increase of pro-inflammatory myeloperoxidase activity and penetrating bacteria were observed only in the ileal tissues of SAMP mice [45].

### 3.4. Inorganic Nanoparticles

Inorganic nanoparticles are often used as additives in dietary products, as well as in pharmaceutical formulations and personal care products [59,60]. In particular, their application in food industry includes improvement of food quality and storage, as well as inhibition of pathogen proliferation. Therefore, it is easy to assume that increased consumption of processed foods can associate with an increased intake of nanoparticles.

#### 3.4.1. Food Colorants

In the later years, titanium dioxide (TiO_2_) has attracted the attention of the scientific community, as it is one of the most widely used food colorants. Studies aimed at evaluating its potential negative impact on human health are still limited. Subrata Ghosh and collaborators investigated the impact of the food grade microparticles TiO_2_ and aluminosilicates on macrophages isolated from CD patients and healthy controls [46]. Dose-response experiments showed no effect of the microparticles alone on macrophage phagocytosis in both groups. However, high doses of microparticles synergized with bacterial antigens to boost IL-8, tumor necrosis factor-α (TNF-α) and IL-10 production by macrophages, as well as to impair their TGF-β secretion/phagocyte activity [46]. More recently, using a model of experimental colitis, Ruiz et al. reported exacerbation of intestinal inflammation in mice fed a TiO_2_-enriched diet [47]. In particular, it was shown a marked change in the microbiota composition, together with enhanced release of ROS and NLRP3 inflammasome activation, a molecular platform able to detect pathogen- and danger-associated molecular patterns and to induce the maturation of pro-inflammatory cytokines (i.e., pro-IL-1β and pro-IL-18) [47]. Indeed, mice given TiO_2_ had increased production of IL-1β and IL-18 cytokines, alteration of epithelial barrier permeability and consequent bacterial translocation [47]. These observations were further confirmed in rats, in which TiO_2_ nanoparticles were seen to cross the gut epithelial barrier and accumulate in Peyer’s patches and liver after only 7 days of oral exposure [48]. The presence of TiO_2_ nanoparticles in Peyer’s patches associated with increased amount of resident dendritic cells and decreased number of regulatory T cells [48]. These observations raise the possibility that foods containing such a particle may compromise gut tolerance towards food antigens and commensal bacteria. Moreover, prolonged oral intake of TiO_2_ nanoparticles (100 days) promoted intestinal low-grade inflammation and consequent colon carcinogenesis in exposed rats [48]. More recently, Mu et al. reported an impairment of the probiotic taxa, such as *Bifidobacterium* and *Lactobacillus*, together with a worsening of dextran sodium sulfate-induced colitis in weaned mice exposed to TiO_2_ nanoparticles for 16 weeks [49]. Mice orally given TiO_2_ exhibited decreased amount of some healthful bacterial metabolites, such as short-chain fatty acids, and increased production of trimethylamine, which has been linked to atherosclerosis development [50]. In addition, dietary TiO_2_ dissolved in drinking water led to a decrease in mucus-related gene expression (i.e., *Muc-2*) and consequent impairment of the intestinal epithelial barrier, an increase in the antimicrobial peptide *Defb3* gene expression, enhanced inflammatory response, and alteration of colonic crypt length [50]. A recent study by Mao and colleagues also investigated the impact of the exposure to TiO_2_ particles in pregnant rats [51]. The authors reported a slight change in gut microbiota composition at gestation day 10 and 17 [51]. In addition, fasting blood glucose levels, together with the expression of diabetes mellitus-related genes, were increased in the exposed rats [51]. Altogether, such observations suggest that TiO_2_ particles may exert detrimental effect also in pregnant women.

TiO_2_ can also be found in food products in combination with other nanoparticles, such as nano-structured synthetic amorphous silica (SAS), which are often used as thickening agent and or foaming controller [61]. Both TiO_2_ and SAS nanoparticles activate NLRP3 inflammasome in lipopolysaccharide (LPS)-primed bone marrow-derived dendritic cells and induce IL-1β cytokine release through cell apoptosis and ROS production [52].

#### 3.4.2. Antimicrobial Agents

Silver nanoparticles (AgNPs) are widely used during food processing for their efficient antimicrobial activity. Shahare and collaborators showed that AgNPs (3–20 nm in size) were able to damage intestinal microvilli and glands in the small intestine, cross the intestinal epithelial barrier and decrease body weight of treated mice [53]. Notably, such effects were seen after 21 days of oral AgNPs administration in a dose-dependent manner (highest weight loss was achieved at the dose of 10 mg/Kg) [53]. However, it is noteworthy that the effects of AgNPs on gut homeostasis would seem to be strictly dependent on particle size, dosage and administration route. Indeed, mice fed with a lower dose (46, 460 or 4600 part per billion) of bigger AgNPs particles ( ~ 55 nm) for 28 days exhibited only intestinal dysbiosis, associated with changes in α- and β-diversity of microbial species, but neither intestinal damage nor change in the expression of inflammatory markers [54]. Gut dysbiosis induced by a short-time exposure of male rats to both sphere- and cube-shaped AgNPs associates with the development of anxiety-like behaviours even though the effect is more evident in mice treated with spherical AgNPs [55]. However, no gut and/or brain histopathological alterations are usually seen in mice receiving AgNPs [55].

Another commonly applied agent is the cationic homopolymer ε-polylysine, certified as GRAS (Generally Recognized as Safe) and used in food processing in United States, Korea and Japan. Daily intake of ε-polylysine for 1, 5 and 9 weeks at the dose of 1.4 × 10^−6^ g/g body weight in mice, which corresponds to the U.S. average per capita annual consumption of soft drinks, perturbs transiently both composition and function of gut microbiome [56]. Although the initial microbiota can be re-established at the end of the ε-polylysine-enriched-diet [56], this observation raises the possibility that prolonged intake of this agent may trigger permanent alterations of microbial composition and induce colitis.

Finally, it is important to point out that intake of anti-microbial agents can also occur through the usage of cosmetics and personal care products. An example is given by triclosan (TCS), a biocidal additive found in several products, such as toothpastes, cosmetics and toys [62]. Although the use of TCS as food preservative was banned in Europe Union and United States, people might be regularly exposed to this agent, both superficially, through skin contact with cream and/or toys, and systemically, via accidental swallowing of toothpaste. Indeed, TCS was detected in urine samples collected from volunteers in the United States [63]. Exposure to low-doses of TCS (10 and 80 parts per million in diet) promotes low-grade intestinal inflammation, colitis and colitis-associated colon carcinogenesis in mice [57]. The mechanisms underlying these effects are dependent on the ability of TCS to promote gut dysbiosis and trigger the signalling cascade downstream the Toll-like receptor 4, a LPS recognizing receptor whose activation promotes intestinal inflammatory responses [57].

### 3.5. Future Perspective

The food additive market size is expected to growth significantly in the next years. Thus, the need to precisely figure out both type and amount of the additives commonly present in the food, combined with data regarding their predicted daily intake, is an urgent aspect that competent agencies should consider in order to re-evaluate their safety. Last year, the Office of Food Additive Safety/Center for Food Safety and Applied Nutrition from FDA published a study aimed at calculating dietary exposure to seven emulsifiers commonly used in the United States for their safety re-assessment [64]. The research was conducted in response to results published by Chassaing and colleagues documenting the detrimental effects of the dietary emulsifiers P80 and CMC on gut homeostasis [33]. Exposure to these compounds was calculated on the American population (starting from 2-year-old subjects) from 1999 to 2002 and from 2003 to 2010, taking into account data sources from the National Health and Nutrition Examination Survey and NPD Group, Inc.’s National Eating Trends—Nutrient Intake Database [64]. Results showed that Americans are particularly exposed to lecithin and diglyceride, whereas P80 and CMC exposure dose is very low, suggesting that concerns about their safety is unfounded as the levels estimated are far from those used by Chassaing and colleagues in their study [64]. Giving as correct the per capita daily intake estimation reported by Shah and colleagues, the study still does not reflect the current and future scenario of food additive consumption in Westernized countries [64]. In fact, it was a retrospective study covering a defined period until 2010 [64], whereas the food additive market has been rapidly growing since that time. Nevertheless, it represents one of the first reports aimed at filling this lack of knowledge and additional studies addressing this aspect for other food additives are needed. In this regard, the EFSA is already conducting a safety re-evaluation procedure of all food additives authorised by European Union prior to 2009, whose completion is expected by the end of 2020 [65]. Results from this investigation will hopefully help to figure out new maximal dose and exposure limits for public health.

## 4. Conclusions

The use of food additives has rapidly expanded worldwide during the last decades and is expected to further increase in the future. In recent years, an accumulating number of studies reported detrimental effects of some commonly used food additives on gut homeostasis, suggesting a link between their consumption and the development/worsening of human intestinal and metabolic diseases. Since most of the studies published so far have been performed in animals, it would be relevant to ascertain whether similar effects occur also in human beings before drawing any conclusion on the deleterious effect of additives on gut homeostasis.

## Figures and Tables

**Table 1:** Effects of food additives on gut homeostasis. All the indicated substances were used in pre-clinical studies and administered orally. All the food additives were dissolved in drinking water unless otherwise indicated in the “Experimental dosage”.

Food Additive	Category	Effect on Gut Homeostasis	Experimental Dosage	Reference
CMC/P80 ADI: GRAS; used in food up to 2%	Emulsifier	Dysbiosis and metabolic syndrome; colitis in *Il10*^-/-^ and *Tlr5*^-/-^ mice; low-grade intestinal inflammation in wild-type mice.	1% *w*/*v*	[33]
		Colorectal cancer	1% *w*/*v*	[34]
		Expansion of human pro-inflammatory bacteria	1, 0.5, 0.25 or 0.10%	[35]
		Sex-specific behavioural and neural alterations in mice	1% *w*/*v*	[36]
MDX ADI: GRAS	Coating and thickening agent	Necrotizing enterocolitis in preterm piglets	47 or 55.2 g/L	[37]
		Outgrowth of AIEC strain	0.4% in medium	[38]
		Impaired response to *Salmonella* infection	5% *w*/*v*	[39]
		Decreased mucus production, increased susceptibility to colitis, low-grade intestinal inflammation	5% *w*/*v*	[40]
NAS ADI: Saccharine: 15 mg/Kg Sucralose: 5 mg/Kg Aspartame: 40 mg/Kg	Non-caloric artificial sweetener	Intestinal dysbiosis and glucose intolerance	Saccharine: 5%; Sucralose 5%; Aspartame 4%	[41]
		Alteration of host microbiota and related metabolites; liver inflammation.	0.1 mg/mL	[42]
		Alteration of microbiota composition and related metabolites of mouse pups; impairment of their hepatic detoxification mechanisms.	0.2 mg/20 µL	[43]
Neotame ADI: 0.3 mg/Kg	Non-caloric artificial sweetener	Intestinal dysbiosis; increased secretion of cholesterol and lipid in faeces.	0.75 mg/Kg by oral gavage	[44]
Splenda ADI: 3.5 mg/Kg	Non-caloric artificial sweetener	Intestinal dysbiosis and intestinal inflammation; *Proteobacteria* expansion and increased ileal myeloperoxidase activity.	1.08–3.5 mg/mL	[45]
TiO_2_ ADI: levels must not exceed 1% of the food weight	Food colorant	Increased cytokine production and impaired phagocyte activity (at the higher dose).	0.05–50 µg/mL	[46]
		Intestinal inflammation and dysbiosis, ROS release, NLRP3 inflammasome activation and IL-1β and IL-18 cytokine release; increased intestinal permeability	10-50-500 mg/Kg by oral gavage	[47]
		Accumulation in Peyer’s Patches; higher frequency of resident DCs and decreased number of regulatory T cells	10 mg/Kg	[48]
		Impairment of the probiotic taxa (*Bifidobacterium* and *Lactobacillus*), together with a worsening of DSS-induced colitis	0.1% in food	[49]
		Alteration of bacterial metabolites; decrease in mucus-related gene expression (i.e., *Muc-2*) and an increase in the antimicrobial peptide *Defb3* gene expression; inflammatory response and alteration of colonic crypt length	2-10-50 mg/Kg	[50]
		Increased fasting blood glucose levels and expression of diabetes mellitus-related genes in pregnant rats	5 mg/Kg	[51]
SAS ADI: GRAS	Thickening agent/foam controller	NLRP3 inflammasome activation in DCs and IL-1β cytokine release upon apoptosis and ROS production.	20 or 40 µg/cm^2^ in medium	[52]
AgNPs ADI: GRAS	Antimicrobial agent	Intestinal microvilli and gland damage, body weight loss, intestinal dysbiosis	46-460-4600 ppb in food	[53,54,55]
		Development of anxiety-like behaviours	3.6 mg/Kg	[55]
ε-Polylysine ADI: GRAS	Antimicrobial agent	Intestinal dysbiosis.	1.4 × 10^−6^ g/ g body weight	[56]
TCS ADI: GRAS, banned from soap.	Antimicrobial agent	Intestinal dysbiosis, low-grade intestinal inflammation and colitis-associated colon carcinogenesis.	5-10-80 ppm	[57]

Abbreviations: ADI: acceptable daily intake; AgNPs: silver nanoparticles; AIEC: adherent invasive *E. coli*; CMC: carboxymethylcellulose; DCs: dendritic cells; Defb3: defensin beta 3; DSS: dextran sodium sulfate; GRAS: generally recognized as safe; IL: interleukin; MDX: maltodextrin; Muc-2: mucin-2; NAS: non-caloric artificial sweetener; NLRP3: NACHT, LRR and PYD domains-containing protein 3; P80: polysorbate 80; ROS: reactive oxygen species; SAS: synthetic amorphous silica; TCS: triclosan; TiO2: titanium dioxide; TLR: toll-like receptor.
